# Targeting hypoxia-induced tumor stemness by activating pathogen-induced stem cell niche defense

**DOI:** 10.3389/fimmu.2022.933329

**Published:** 2022-09-29

**Authors:** Seema Bhuyan, Bidisha Pal, Lekhika Pathak, Partha Jyoti Saikia, Shirsajit Mitra, Sukanya Gayan, Reza Bayat Mokhtari, Hong Li, Chilakamarti V. Ramana, Debabrat Baishya, Bikul Das

**Affiliations:** ^1^ Department of Cancer and Stem Cell Biology, KaviKrishna Laboratory, Research Park, Indian Institute of Technology, Guwahati, India; ^2^ Department of Bioengineering and Technology, Gauhati University, Guwahati, India; ^3^ Department of Stem Cell and Infectious Diseases, KaviKrishna Laboratory, Research Park, Indian Institute of Technology, Guwahati, India; ^4^ Department of Immunology and Infectious Diseases, Forsyth Institute, Cambridge, MA, United States; ^5^ Department of Experimental Therapeutics, Thoreau Laboratory for Global Health, M2D2, University of Massachusetts, Lowell, MA, United States

**Keywords:** Stem cell niche, Tumor stemness, Bacillus calmette guerin, Tumor hypoxia and oxidative stress microenvironment, Cancer Stem Cells (CSCs), Altruistic Stem Cells (ASCs)

## Abstract

Tumor hypoxia and oxidative stress reprograms cancer stem cells (CSCs) to a highly aggressive and inflammatory phenotypic state of tumor stemness. Previously, we characterized tumor stemness phenotype in the ATP Binding Cassette Subfamily G Member 2 (ABCG2)–positive migratory side population (SPm) fraction of CSCs exposed to extreme hypoxia followed by reoxygenation. Here, we report that post-hypoxia/reoxygenation SPm+/ABCG2+ CSCs exerts defense against pathogen invasion that involves bystander apoptosis of non-infected CSCs. In an *in vitro* assay of cancer cell infection by Bacillus Calmette Guerin (BCG) or mutant *Mycobacterium tuberculosis (Mtb) strain 18b (Mtb-m18b)*, the pathogens preferentially replicated intracellular to SPm+/ABCG2+ CSCs of seven cell lines of diverse cancer types including SCC-25 oral squamous cancer cell line. The conditioned media (CM) of infected CSCs exhibited direct anti-microbial activity against Mtb and BCG, suggesting niche defense against pathogen. Importantly, the CM of infected CSCs exhibited marked *in vitro* bystander apoptosis toward non-infected CSCs. Moreover, the CM-treated xenograft bearing mice showed 10- to 15-fold reduction (p < 0.001; n = 7) in the number of CSCs residing in the hypoxic niches. Our *in vitro* studies indicated that BCG-infected SPm+/ABCG2+ equivalent EPCAM+/ABCG2+ CSCs of SCC-25 cells underwent pyroptosis and released a high mobility group box protein 1 (HMGB1)/p53 death signal into the tumor microenvironment (TME). The death signal can induce a Toll-like receptor 2/4–mediated bystander apoptosis in non-infected CSCs by activating p53/MDM2 oscillation and subsequent activation of capase-3–dependent intrinsic apoptosis. Notably, SPm+/ABCG2+ but not SP cells undergoing bystander apoptosis amplified the death signal by further release of HMGB1/p53 complex into the TME. These results suggest that post-hypoxia SPm+/ABCG2+ CSCs serve a functional role as a tumor stemness defense (TSD) phenotype to protect TME against bacterial invasion. Importantly, the CM of TSD phenotype undergoing bystander apoptosis may have therapeutic uses against CSCs residing in the hypoxic niche.

## Introduction

Cancer stem cells (CSCs) are endowed with self-renewal capacity and reside in specific niches in the tumor microenvironment (TME). CSCs reside in both the perivascular and hypoxic niches and maintain a steady proportion among the heterogeneous cancer cell population (1). CSCs are found to express drug efflux pumps such as ATP Binding Cassette Subfamily G Member 2 (ABCG2) (1), exhibiting marked detoxification and antioxidant activities that make them inherently resistant to chemotherapy and radiation therapy. Moreover, targeted therapies that inhibit oncogene or growth factor–driven pathways fail to target CSCs (2), as these self-renewing cancer cells may activate multiple growth factor pathways. Anti-angiogenesis therapy may aid into the CSC maintenance by increasing tumor hypoxia (3). Furthermore, immunomodulatory attributes of CSCs make them exceptionally adept at evading immune monitoring (4) and resistant to immunotherapy. Thus, CSCs are difficult to target by conventional anti-cancer strategies.

Importantly, hypoxia and oxidative stress prevailing in TME may reprogram CSCs to a highly inflammatory and aggressive phenotype of tumor stemness (TS) switch (5–7). Moreover, chemotherapy- and radiation-induced oxidative stress may also reprogram CSCs to this TS phenotype (8). Earlier, we reported a cisplatin-mediated TS phenotype in migratory side population (SP) cells; following cisplatin therapy, these cells exhibited rapid self-renewal, expressed stemness genes such as octomer-binding transcription factor 4 (Oct-4) and Nanog as well as secreted angiogenic growth factors (8). Others also reported drug-induced stemness in several tumor models (9, 10). Subsequently, using a SCC-25 oral cancer–derived CSC model, we showed that hypoxia/reoxygenation or chemotherapy-induced TS enables ABCG2-positive sub-fraction of EpCAM+/ALDH1+/CD44v3+ CSCs to modulate TME for rapid tumor progression and immune suppression (11, 12). In addition, we found that oral cancer EpCAM+/ABCG2+ cells undergo inflammation and bacteria-mediated TS phenotype (13), which also contribute to rapid tumor progression. Recently, the inflammatory and aggressive component of CSCs was identified in several cancers including colorectal cancer (14). The TS phenotype may be part of the CSC niche defense mechanism to maintain the stemness state of CSCs (5–7) and, therefore, can be termed as a TS defense (TSD) phenotype. Therefore, there is a strong rationale to develop innovative therapies to target the CSC-activated TSD phenotype.

To explore novel ways to target CSCs residing in the hypoxic niches, we revisited our work on embryonic and mesenchymal stem cell niche defense against oxidative stress and pathogens that involve bystander apoptosis. Stem cell resides in their secured niches, often in a hypoxic, and immuneprivileged microenvironment to maintain their quiescence state. In these protective niches, pathogens and toxic-free radicals may invade. Therefore, a unique niche defense may have evolved to protect the quiescence stem cells in their niches (15–17). To study this possibility, we developed *in vitro* and *in vivo* models of *Mycobacterium tuberculosis* (*Mtb*) invasion in bone marrow stem cell niche. Further study showed that pathogen-induced alveolar stem cell niche defense involved an altruistic strategy, where the infected lung mesenchymal stem cells undergo an altered p53/MDM2 oscillation pattern to transiently suppress p53 and reprogram to a highly cytoprotectant state, the Altruistic stem cell (ASC) state (16). These ASCs secrete not only antioxidants such as glutathione but also yet unknown anti-microbial agents. Importantly, after a 2-week period of rapid expansion, ASCs undergo spontaneous apoptosis as the p53/MDM2 oscillation returns to basal state. Thus, the ASC-based niche defense mechanism may involve not only secreting anti-microbial factors but also eliminating self to avoid being attractive site for pathogen’s replication. Moreover, non-infected ASCs also undergo apoptosis, suggesting that infected ASCs may secrete soluble factors to activate pathogen-induced bystander apoptosis (PIBA), an innate immune defense mechanism (16). Our work on *Mtb*-infected MSC model indicates that PIBA may be part of the stem cell niche defense, an innate defense mechanism that involves stem cell altruism (16, 17).

Hence, we speculate that this stem cell niche defense–associated PIBA could be utilized to target CSCs of TSD phenotype in the TME niche. Here, we tested this hypothesis by using an *in vitro* model of *Mtb-* and *BCG*-infected CSCs of TSD phenotype. BCG is an attractive candidate for PIBA of CSCs, as it is already in clinical use against bladder cancer for the last four decades. The conditioned media (CM) of BCG-infected CSCs of TSD phenotype (henceforth known as BCG-CM) were used to investigate PIBA.

We found that *BCG* preferentially infects and replicates intracellular to CSCs exhibiting TSD phenotype obtained from diverse cancer cell lines including SCC-25 oral cancer cell line (18). BCG-infected CSCs with TSD phenotype undergo pyroptosis and release a death signal, the high mobility group box protein 1 HMGB1/p53 complex in the BCG-CM, which then induces bystander apoptosis of CSCs in a Toll-like receptor (TLR) 2/4–dependent manner. Whereas CSCs without TSD phenotype did not allow either the pathogen to replicate intracellularly or subsequent release of death signal. These results identify a novel mechanism of CSC niche defense having potential therapeutic implications.

## Materials and methods

### Bacterial strains and culture

All the necessary experimental procedures were undertaken inside BSC class II facility in accordance with the guidelines of “Institutional Bio-safety Committee” of KaviKrishna Laboratory. BCG strain (ATCC^®^ 35737™) was obtained from the American Type Culture Collection (ATCC) and grown in Difco™ Middlebrook 7H9 broth (Becton Dickinson). The media contained 0.5% glycerol, 0.06% Tween 80, and 10% oleic acid albumin dextrose catalase (OADC) (BD, USA). Streptomycin-auxotrophic mutant *Mtb strain18b* (gifted by Prof. Stewart T. Cole, EcolePolytechinqueFederale de Lausanne, Lausanne, Switzerland) was cultured in 7H9 medium (Difco, BD Biosciences, Franklin Lakes, NJ, USA) supplemented with Middlebrook albumin-dextrose-catalase, 0.5% glycerol, and 0.06% Tween 80 (Sigma, St. Louis, MO, USA) until an Optical Density (OD) of around 1 was obtained. We added streptomycin sulfate (50μg/ml) into the *Mtb-m18b* culture medium for bacterial growth. Green fluorescent protein (GFP) tagging procedure was performed as described earlier. The bacteria were prepared as single-cell suspension in RPMI media as previously described (16), being used to infect CSCs.

### Cancer cell culture and sorting of CSCs with TSD phenotype

SKN-BE-2, HOS, RH4, H-146, LOVO, MCF-7, Jurkat, THP-1, and SCC-25 cell lines from ATCC (CRL-1628; Manassas, VA, USA) were maintained as previously described (1, 5, 7). The SCC-25 cells were cultured in Dulbecco’s modified Eagle’s medium containing Ham’s F12 (DMEM F-12) in the ratio of 1:1. DMEM F-12 is enriched with sodium bicarbonate (1.2 g/L), 2.5 mM L-glutamine, 15 mM (4-(2-hydroxyethyl)-1-piperazineethanesulfonic acid (HEPES), and 0.5 mM sodium pyruvate (catalog no. 11330-057, Gibco). The medium was supplemented with hydrocortisone (400ng/ml; catalog no. H0888, Sigma) and 10% fetal bovine serum (catalog no. 16000-044) and used as complete isolation media. The cells were maintained in a humidified atmosphere of 5% CO_2_ at 37°C (11, 12), and the other cell lines were also maintained as previously described (5). Hypoxia/oxidative stress (<0.1% O_2_) was generated in a sealed container using BBL GasPak Plus anaerobic system enveloped with a palladium catalyst (Becton Dickinson, Cockeysville, MD, USA) as previously described (5). To obtain TSD phenotype of CSCs, SP cells were flow cytometry–sorted (5) and then exposed to 24 h of hypoxia followed by 72 h of reoxygenation. Then, migratory SP (SPm) and non-migratory SP (SPn) cells were collected as previously described (5, 6). The post-hypoxia SPm cells or SPm (hox) cells exhibit TSD phenotype and highly express ABCG2 (5, 6). For the SCC-25 cell-derived EpCAM+/ABCG2+ and ABCG2− CSCs, the post-hypoxia/oxidative stress–treated cells ALDH+ cells (Aldefluor kit, #01700, Stem Cell Technologies, Vancouver, BC) were first subjected to immunomagnetic sorting for EpCAM+ cells by using EpCAM antibody (#ab213500, Abcam) conjugated with Fluorescein isothiocyanate (FITC) by a SiteClick antibody labeling kit. This EpCAM+ cells were then expanded for 7 days in spheroidal culture media [serum-free culture containing Epidermal Growth Factor (EGF) and Basic Fibroblast Growth factor (bFGF) (20 ng/ml)] as previously described (5, 7, 18). The EpCAM+/ABCG2+ CSCs were then immunomagnetically sorted by using theABCG2 antibody (#ab3380, Abcam), conjugated with PE by a SiteClick antibody labeling kit as previously described (16). For the immunomagnetic sorting, a Phycoerythrin (PE) sorting kit (#18554, Stem Cell Technologies, BC) was used. Note that both EpCAM+/ABCG2+ CSCs and ABCG2− CSCs of SCC-25 cell lines expressed CD44v3, ALDH1, and CD133 equally. Overexpression of TLR 2/4 in the EpCAM+/ABCG2- CSCs were achieved by transfecting pcDNA3 plasmid (Invitrogen) encoded with human TLR2 and TLR4 using JetPEI reagent as described previously (1).

### BCG and *Mtb-m18b* infection of EpCAM+/ABCG2+ CSCs and collection of BCG-CM

The post-hypoxia/reoxygenation SPm+/ABCG2+ cells or EpCAM+/ABCG2+ immunomagnetically sorted cells were cultured *in vitro* for 48 h and then treated with BCG or *Mtb*-m18b with Multiplicity of Infection (MOI) 5:1 as previously described including treatment with amikacin (200 µg/ml) to kill extracellular bacteria (16). The infected cancer cells were then washed twice with serum-free RPMI and incubated in the appropriate cell culture media for the desired time at 37°C and 5% CO_2_. The CM was collected at desired time starting from day 10 by adding 1 ml of fresh serum-free DMEM per 1 × 10^5^ cells for 48 h and filter-sterilized with 0.2-µm filter, concentrated with Centricon centrifugal filter units (EMD Millipore) to 10× to prepare 0.1 ml of CM containing protein (100 ng/ml). The CM was then utilized to treat fresh EpCAM+/ABCG2+ CSCs to evaluate bystander apoptosis. To collect rifampicin (RIF)–treated CM, the day 9 infected cells were treated with RIF (2µg/ml) for 3 days to kill intracellular bacteria as previously described.

### *In vivo* tumorigenicity assay

All the necessary experimental procedures were undertaken in accordance with approvals of Institutional Animal Ethics Committee of KaviKrishna Laboratory and Gauhati University. To generate subcutaneous tumors, 1 × 10^5^ EpCAM+/ABCG2+ CSCs of SCC-25 cells were mixed with Matrigel (100 µl) and then injected subcutaneously to Nonobese diabetic/severe combined immunodeficiency (NOD/SCID) mice following proper ethical permission as described (19). After a week, mice were injected intraperitoneal with cisplatin 10mg/kg once weekly for two weeks (19) to induce cisplatin-mediated tumor stemness (8). After 6 weeks, when the tumor reached the size of 0.5 mm^3^, the animals were locally injected with concentrated BCG-CM/week (2 ml concentrated to 0.1 ml containing 0.5 mg of protein) into the tumor. The tumor size was measured with a caliper on a biweekly basis for 10 weeks, and tumor volume was determined using the formula 0.5ab^2^, where b is the smaller of the two perpendicular diameters as described (5, 19). After 10 weeks, tumors were dissociated, and single-cell suspension was obtained to perform clonogenic assay and to evaluate the frequency of EpCAM+/ABCG2+ CSCs. For the TLR2/4 neutralizing experiment, BCG-CM was pre-treated with anti-TLR 2/4 antibodies (10 µg/ml each) 4 hours before intra-tumoral injection.

### Clonogenic assay

The single-cell suspension of dissociated tumors was freshly sorted *via* immunomagnetic sorting, and 1 × 10^3^ EpCAM+/ABCG2+ or EpCAM+/ABCG2− CSCs were seeded in methylcellulose medium (Methocult M3134, Stem Cell Technologies) as previously described (1, 5). The cells were seeded in six-well plates, incubated at 37°C and 5% CO_2_. The colonies were counted after 2 weeks (1).

### Real-time PCR

The real-time PCR was performed as previously described using the TaqMan Gene expression assay (1). The glyceraldehyde 3-phosphate dehydrogenase (GAPDH) was used as an endogenous control, and RNA was quantified by the delta-delta CT method using Q-Rex software version 1.1 (Rotor-Gene Q-Qiagen, New Delhi, India). The following TaqMan gene expression primers were used: human: ABCG2 (Hs00184979_m1), TLR2 (Hs02621280_s1), TLR4 (Hs00152939_m1), TLR7 (Hs01933259_s1), TLR9 (Hs00370913_s1), p53 (Hs01034249_m1), p21 (Hs00355782_m1), PUMA (Hs00248075_m1), Bax (Hs00180269_m1), GAPDH (Hs00266705_g1), MDM2 (Hs01066930_m1), and HMGB1 (Hs01923466_g1).

### Pyroptosis assay

The pyroptosis of BCG-infected EpCAM+/ABCG2+ CSCs was evaluated by measuring cleaved caspase-1 level by enzyme-linked immunosorbent assay (ELISA), caspase-1 activity, and lactate dehydrogenase (LDH) release assay. The caspase-1 activity assay was performed by using the caspase-1 substrate Ac-YVAD-AFC (Cayman Chemical, An Arbor, MI, USA) as previously described (20). Briefly, cell lysate prepared for the caspase-3/7 assay was mixed with Ac-YVAD-AFC, and after an hour, the fluorescence signal of cleaved AFC was detected at 400-nm excitation and 505-nm emission using fluorescence spectrofluorometer (Agilent Varian Cary Eclipse, Hyderabad, India). For the LDH release assay, the BCG-CM was subjected to LDH measurement by the LDH-cytotoxicity assay kit (#ab65393, Abcam) as per the manufacturer’s instruction with slight modifications. Briefly, 25 µl of BCG-CM was mixed with 25 µl of LDH assay reagent, and the assay reaction was stopped after 30 min by adding 25 µl of stop solution. The OD value at 450 nm was taken using iMarkMicroplate Absorbance Reader (Bio-Rad, Gurgaon, India). Some of the assay results were confirmed by the Decker method (21). To further confirm pyroptosis, LDH was also measured after treating the cells with disulfiram 50 nM/twice daily or caspase-1 inhibitor z-YVAD-fmk.

### Cellular apoptosis or caspase-3/7 activity assay

The assay was performed as previously described by using the caspase-3/7 substrate Ac-DEVD-AMC (1, 5, 22). Briefly, cell lysate (100 µg/ml) was prepared by lysing 5 × 10^3^ to 1 × 10^5^ cells using a modified 1× RIPA buffer: Tris-HCl (20 mM; pH 7.5), NaCl (155 mM), 1 mM Na_2_ Ethylenediamine tetraacetic acid (EDTA) (1 mM EDTA from 100 mM stock solution in H_2_O; pH 7.4), Ethylene glycol-bis (β-aminoethyl ether)-N, N, N'-tetraacetic acid) (EGTA) (1.5 mM EGTA), Triton (1.2%), sodium pyrophosphate (25 mM), sodium fluoride (25 mM), β-glycerophosphate (1 mM), activated sodium orthovanadate (Na_3_VO_4_), 1 mM (from 200 mM stock solution), leupeptin (1 µg/ml), aprotinin (1 µg/ml), and pepstatin (1 µg/ml). Phenylmethylsulfonyl fluoride (PMSF; 1 mM) (200 mM stock solution prepared in isopropanol and stored at RT) and dithiothreitol (DTT; 5 mM) were added immediately before use. Cells in a 1.5-ml microcentrifuge tube were centrifuged in ice-cold phosphate-buffered saline (PBS), and the pellet was treated with 50 µl of RIPA buffer, kept on ice for 10 min, and stored at −80°C. After a few days, lysate was thawed on ice; equal volume of freshly prepared RIPA buffer was added, vortexed for 1 min, kept on ice for 5 min, and then centrifuged at 4°C/5,000 RPM; and the supernatant was transferred to a fresh tube as 25 µl of aliquot and stored at −80°C for future use. To perform the assay, 25 µl of aliquot was thawed on ice, and 200 µl of Ac-DEVD-AMC (Cayman Chemical, An Arbor, MI, USA) substrate (prepared by adding 0.1 ml of 1 mg of Ac-DEVD-AMC in DMSO to 4 ml of the lysis buffer containing freshly added DTT and PMSF) was added on to it. The mixture was vortexed, and the enzymatic activity was measured by detecting cleaved substrate linked to fluorophore using a fluorescence spectrofluorometer (Agilent Varian Cary Eclipse, Hyderabad, India), as previously described.

### Caspase inhibition

The experiment was performed as previously described (1, 5). Anti–caspase-8 is the Z-IETD- FMK (fluoromethyl ketone; R&D Systems, #FMK007), and anti–caspase-9 is Z-LEHD-FMK (#FMK 008). For both caspase inhibition experiment, 100 µM (dissolved in DMSO) of each was added in the cell culture for 4 days. The medium was changed every second day.

### Inhibition of ferroptosis, necroptosis, and autophagy

Ferrostatin-1 (20 µM), necrostatin or RIP-I kinase (20 µM), and 3-methyladenine (3-MA) of 5 mM was prepared in DMSO (from 100 mM stock). These reagent mixtures were used to inhibit ferroptosis, necroptosis, and autophagy, respectively. Reagents were obtained from Sigma-Aldrich and neutralizing HMGB1 (10 µg/ml) was obtained from BioLegend (isotype control mouse IgG2a kappa; #16-4724, BioLegend).

### Enzyme-linked immunosorbent assay

The cell lysates were prepared by RIPA buffer and subjected to ELISA assay as described (16). The information about various ELISA kits and antibody details is given in the **Supplementary Method** and **Table 1**. The absorbance was measured at 450 nm using the iMarkMicroplate Absorbance Reader (Bio-Rad, Gurgaon, India).

### Western blot and co-immunoprecipitation

Western blot (WB) analysis was done on a 4%–12% sodium dodecyl sulfate–polyacrylamide gel electrophoresis gel and transferred to polyvinylidenedifluoride membranes (Immobiolon-P, MilliporeSigma, cat. # IPVH20200), as previously described (1, 5). Co-immunoprecipitation (co-IP) of the HMGB1-p53 complex in the BCG-CM was performed following the laboratory’s standard IP protocol (1) using the protein A sepharose beads (GE, MilliporeSigma, cat. # 17-0780-01). The concentrated BCG-CM (containing 1.0 mg of protein in the lysis buffer) was subjected to cross-linking by 3 3'-dithiobis-sulfosuccinimidyl propionate (DTSSP) (Thermo Fisher Scientific, #21578) as per the manufacturer’s instructions before performing IP. Prior to IP, samples were pre-cleared with Protein A Sepharose at 4°C for 3 h with gentle shaking. A rabbit polyclonal antibody (10 µg; #ab228624, Abcam) was used to allow HMGB1 immune complex to form (in 500 µl of solution containing 1.0 mg of protein), which was captured with 50% slurry of Bovine serum albumin (BSA) blocked Protein A Sepharose beads. The immune complex was then eluted by boiling the beads in 2× SDS sample buffer, and the elutes were washed four times with 1× PBS with 0.2% Tween 20. The elutes were subjected to WB probing with a mouse monoclonal antibody (#H00003146-M08, Novus Biologicals) against human HMGB1. To confirm the p53 IP, blots were stripped (Thermo Fisher Restore stripping buffer, cat. # 21059) and re-probed using p53 antibody (#2527, Cell Signaling Technology). Inputs representing 2.5% of the lysate subjected to IP were further subjected to WB for HMGB1 using the mouse monoclonal antibody. The co-IP elutes (eluted with elution buffer containing glycine and Tris-HCl and 500 mM NaCl) were also subjected to ELISA to quantify p53 and HMGB1 proteins after the elutes were neutralized by 10× PBS. Isotype control with a rabbit polyclonal IgG (ab#37415, Abcam) was run in parallel to the sample in each IP procedure.

### Silencing of p53

The inhibition of p53was achieved by Accell siRNA (Thermo Scientific Dharmacon, Lafayette, CO, USA) and by pifithrin-α, an inhibitor of p53, as previously described (1, 23). The AccellsiRNA used for p53 was A-003329-22-0005. Briefly, the EpCAM+/ABCG2+ CSCs (10^4^ cells per well in 96-well plate) before treating with BCG-CM were treated with 1 μM Accell siRNA as per the manufacturer’s instructions. After 72 h of incubation at 37°C, gene silencing was confirmed using real-time Quantitative Polymerase Chain Reaction (qPCR).

### Antibody blocking experiments

The experiment was performed, as previously described (1, 5), anti-Fas monoclonal antibody (human, neutralizing) clone ZB4 (Sigma-Aldrich); anti-human tumor necrosis factor (TNF)–monoclonal antibody (MAB 210; R&D Systems, Minneapolis, MN, USA), anti-human TRAIL (clone RIK-2; Thermo Fisher Scientific, Waltham, MA, USA); anti-hLAP or transforming growth factor (TGF)–beta 1 (MAB 246; R&D Systems, Minneapolis, MN, USA); anti-TLR2 (cloneTL2.1; BioLegend, San Diego, CA, USA), and anti-TLR4 (HTA125; BioLegend, San Diego, CA, USA). Immunoglobulin G1 (IgG1) isotype control antibodies were used at the corresponding concentrations. Antibodies (5µg/ml) were added to BCG-CM and mixed well before adding to EpCAM+/ABCG2+ CSC grown in six-well culture plates. The abilities of the blocking antibodies to neutralize their ligands were determined by challenging Jurkat or undifferentiated THP-1 cells with the appropriate ligand in the presence of the antibody at the concentrations indicated above. Fas ligand (MAB050), TRAIL (375-TL-010), TGF-beta 1 (7754-BH-005), and TNF-alpha (210-TA) were obtained from R&D Systems, and the synthetic bacterial lipopeptide Pam3CysSerLys4 was obtained from Calbiochem as described (24). The cells were incubated with cycloheximide (0.4 µg/ml) for 15 min before the addition of the apoptotic stimulus, the TNF-alpha or Pam3CysSerLys4 ligands to test TNF-alpha and TLR2/4 induced apoptosis respectively. To validate the neutralizing effects of the antibodies, Jurkat or THP-1 cells were treated with these ligands in appropriate concentrations (Fas ligand 10ng/ml, TRAIL 20ng/ml, TNF-alpha 5 ng/ml, and Pam3CysSerLys4 20 ng/ml) for 48 hours with or without the neutralizing antibodies. Dexamethasone 30 nM was added with TGF-beta ligand (3ng/ml) to test neutralizing effet of anti-TGF-β in THP-1 cells.

### Statistical analysis

The statistical calculations were performed using either Student’s t-test or one-way ANOVA with Dunnet *post hoc* test by GraphPad Prism version 8.4.2). Data are expressed as means ± SEM; *p < 0.05, **p < 0.01,*** *p <* 0.001, and ****p < 0.0001.

## Results

### BCG replicates intracellular to SPm (hox)+/ABCG2+ CSCs and induces pyroptosis

**Figure 1:**
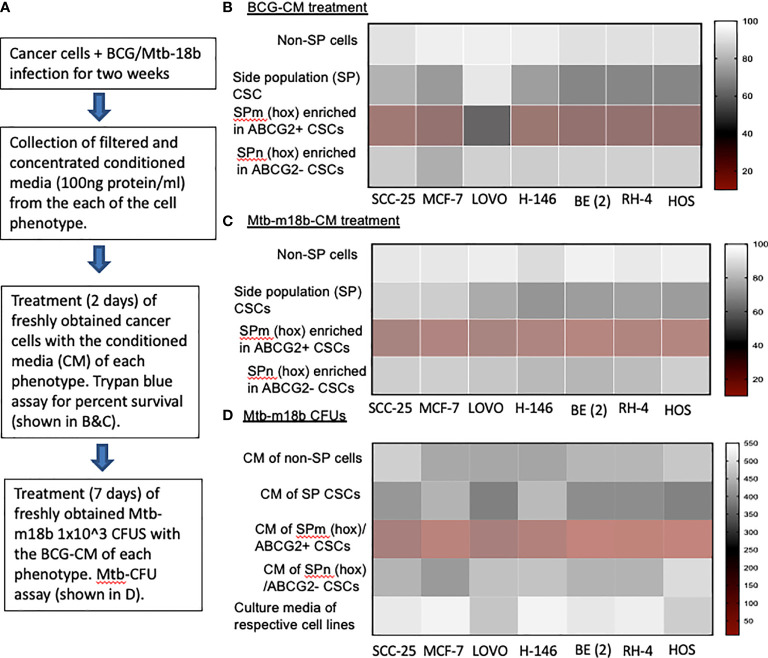
The tumor stemness phenotype of SPm (hox)/ABCG2+ CSCs exhibits BCG- or Mtb-m18b–mediated bystander apoptosis and anti-microbial activity. **(A)** Experimental plan. To investigate the niche defense potential of each phenotype, we treated the conditioned media (CM) of infected phenotype with the untreated corresponding phenotype. **(B, C)** Marked bystander cell death is seen in the SPm (hox) cells enriched in EpCAM+/ABCG2+ CSCs group. These SPm (hox) cells exhibit tumor stemness (TS) phenotype (5–7). **(D)** The CM of SPm(hox)+/ABCG2+CSCs exhibit anti-microbial activity against Mtb-m18b. The vertical bar represents percent of cell survival in **(B, D)**, and number of Mtb-m18b CFUs in **(D)**. Experiments were repeated 4 times, and the results were compared between SPm (hox) and SPn (hox) by student t tests. Results are given in the text.

We hypothesized that BCG and *Mtb* may induce PIBA of CSCs exhibiting TS phenotype, as this phenotype may activate the “stem cell niche defense” mechanism to defend their TME niche similar to ASCs (16, 23, 24). This innate defense mechanism may involve not only secreting anti-microbial factors but also eliminating self to avoid being an attractive site for pathogen’s replication. To test this hypothesis, we have utilized several cancer cell line–derived xenograft models, where we characterized the TS phenotype. In these xenografts of neuroblastoma (SKN-BE-2), sarcoma (HOS and RH4), small cell lung cancer (H-146), colon cancer (LOVO), breast cancer (MCF-7), and oral squamous cell cancer (SCC-25), we found that migratory side population cells (SPm) enriched in ABCG2+ CSCs having high tumorigenic activity reside in the hypoxic TME niche and exhibit TS phenotype (5–7, 11). We previously obtained this highly tumorigenic SPm+/ABCG2+/ABCG2+ CSCs from SP cells (25) when exposed to 24 h of hypoxia followed by 24 h of reoxygenation (5). We termed these cells as post-hypoxia migratory SP cells or SPm (hox) cells (5). These SPm (hox) cells exhibit TS phenotype and enriched in ABCG2+ CSCs (5–7). Whereas post-hypoxia non-migratory SP cells or SPn (hox) cells were enriched in ABCG2− CSCs (5–7). To investigate the niche defense potential of both SPm (hox) and SPn (hox) CSC phenotypes, the CM of these phenotypes obtained from diverse tumor cell lines were collected 2 weeks after infection with *Mtb-m18b* or BCG. The CM was then concentrated and added to freshly obtained cancer cells to investigate PIBA (**Figure 1A**). The CM was added into the freshly obtained Mtb-18b or BCG to investigate the niche defense against invading pathogens (**Figure 1A**). As shown in **Figures 1B, C**, there was a four- to five-fold loss of viability of TS phenotype–enriched SPm (hox)+/ABCG2+ CSCs as compared with post-hypoxia/reoxygenation SPn(hox)+/ABCG2- CSCs (p < 0.001; **Figures 1B, C**) and/or pre-hypoxia SP cells. Importantly, the CM of these SPm(hox)+/ABCG2+ CSCs exhibited three- to four-fold anti-microbial toxicity (p < 0.05; **Figure 1D**). These results suggest that the TS phenotype may defend their niches from pathogen infection by direct anti-microbial activity as well as inducing PIBA of neighbor SPm (hox)+/ABCG2+ CSCs (**Figures 1B, C**).

**Figure 2:**
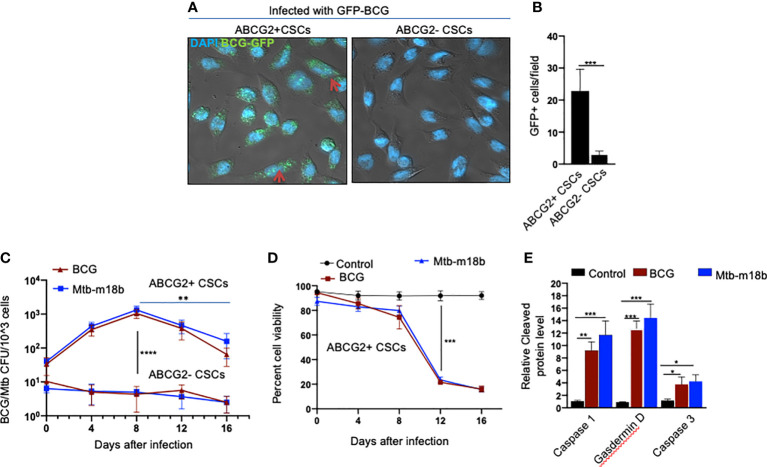
BCG replicates intracellular to EpCAM+/ABCG2+ CSCs of SCC-25 cell line and induces pyroptosis. Post hypoxia/reoxygenation treated EpCAM+/ABCG2+ CSCs (henceforth known as EpCAM+/ABCG2+ CSCs) obtained from SCC-25 cells exposed to the *in vitro* system of hypoxia and reoxygenation (5) is sensitive to BCG-induced cell death. **(A)** Confocal microscopy images (magnification, 20×) showing the localization of GFP-positive BCG intracellular to EpCAM+/ABCG2+ CSCs (shown with arrows) versus post hypoxia/reoxygenation treated EpCAM+/ABCG2- CSCs (henceforth known as EpCAM+/ABCG2- CSCs). **(B)** Histogram shows 20-fold increase in GFP-positive BCG per hundred microscopic field in EpCAM+/ABCG2+ CSCs versus EpCAM+/ABCG2- CSCs. **(C)** Intracellular BCG/Mtb-m18b-CFU in EpCAM+/ABCG2+ CSCs versus EpCAM+/ABCG2- CSCs after infection. **(D)** Trypan blue assay of cell viability of BCG/Mtb-m18b–infected EpCAM+/ABCG2+ CSCs after infection. **(E)** ELISA-based measurement of cleaved caspase-1, gasdermin D, and caspase-3 levels in EpCAM+/ABCG2+ CSCs of day 12 after BCG/Mtb-m18binfection. Data represent means ± SEM **(B–D)**. N = 3 independent experiments; *p < 0.05, **p < 0.01, ***p < 0.001, and ****p < 0.0001 (**B–D**: Student’s t-test; E: one-way ANOVA with Dunnet post hoc test).

We reasoned that exploring the BCG-induced PIBA in oral cancer CSCs may have clinical utility, as oral cancer lesions are externally accessible for future BCG immunotherapy. Hence, we decided to study the potential PIBA in the SCC-25 cell line–derived TS phenotype. Considering that it is challenging to obtain the SPm (hox)/ABCG2+ CSCs, we obtained the equivalent population of EpCAM+/ABCG2+ CSCs (11, 12, 18). Thus, the EpCAM+/ABCG2+ CSCs/ABCG2− CSCs were immunomagnetically sorted from post-hypoxia/oxidative stress–treated EpCAM-positive SCC-25 cells (11, 12, 18). These EpCAM-positive cells expresses other CSC markers including ALDH1 and CD44v3 (11, 12). These post hypoxia/rexoygenation EpCAM+/ABCG2+ CSCs (henceforth known as EpCAM+/ABCG2+ CSCs) obtained from SCC-25 cells exposed to the *in vitro* system of hypoxia and reoxygenation (5) are sensitive to BCG-induced cell death. The sorted CSCs were infected with GFP-tagged BCG and subjected to confocal microscopy. The *Mtb* colony-forming unit (CFU) was performed after 4 days of *in vitro* cell growth of CSCs infected with GFP-tagged BCG. *Mtb*-CFU assay confirmed the internalization and replication of the GFP-BCG mostly to EpCAM+/ABCG2+ CSCs versus EPCAM+/ABCG2– CSCs (**Figures 2A, B**). The result suggests that the BCG pathogen may selectively infect and replicate in EpCAM+/ABCG2+ CSCs versus EpCAM+/ABCG2- CSCs. The intracellular replication of pathogen in EpCAM+/ABCG2+ CSCs versus ABCG2− CSC is not restricted to *Mycobacterium bovis*, as similar result was observed when the CSCs were infected with an *Mtb* strain m18b, and the infected cells were grown for 4 days. This selective uptake of BCG/*Mtb* by EpCAM+/ABCG2+ CSCs, as well as their intracellular replication, allows us to evaluate long-term fate of these infected cells.

In macrophages, *Mycobacteria* are known to replicate during the first week of infection (26), and subsequently, the host cells undergo cell death by apoptosis and pyroptosis by the second week of infection (27). To evaluate whether BCG and *Mtb-m18b*–infected EpCAM+/ABCG2+ CSCs may also undergo cell death by apoptosis and pyroptosis due to intracellular replication of the pathogen, the infected cancer cells (10^4^/ml) were grown *in vitro* for 16 days to find out the day when pathogen replication goes down with associated increase in host cell death. BCG/*Mtb*-*m18b–*infected EpCAM+/ABCG2- CSCs served as control. Thus, every fourth day, 5 × 10^3^ cells were recovered, subjected to trypan blue viability assay, and then lysed to perform the BCG/*Mtb-m18b* CFU assay for evaluating intracellular bacterial replication. Indeed, BCG/*Mtb-*m18b–infected EpCAM+/ABCG2+ CSCs showed a 100-fold (p < 0.0001; **Figure 2C**) increase in the number of intracellular CFUs on day 8 without exhibiting any marked loss in cell viability (**Figure 2D**). These results further confirm that the pathogens selectively infect and replicate in EpCAM+/ABCG2+ CSCs versus EpCAM+/ABCG2- CSCs. Notably, the pathogen-infected EpCAM+/ABCG2+ CSCs showed a marked loss of intracellular CFUs between days 8 and 16 (**Figure 2C**), as well as a 4.5-fold loss of viability between days 8 and 12 (p < 0.001; **Figure 2D**). On day 12, the infected EpCAM+/ABCG2+ CSCs exhibited significant upregulation of caspase-3, as well as caspase-1, and gasdermin D, the markers of pyroptosis (**Figure 2E**). These results indicate that the *Mycobacteria* selectively infect, replicate, and then induce apoptosis/pyroptosis in EpCAM+/ABCG2+ CSCs of SCC-25 cell line.

### The CM of BCG-infected EpCAM+/ABCG2+ CSCs induces bystander apoptosis in non-infected EpCAM+/ABCG2+ CSCs

**Figure 3:**
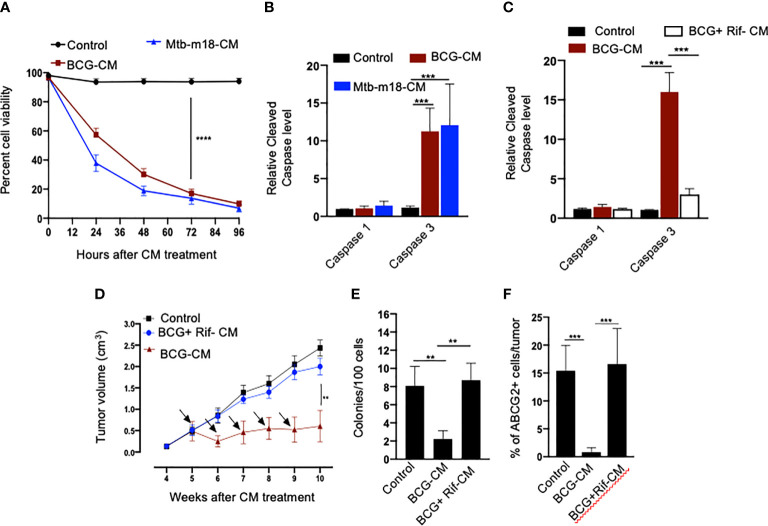
The CM of BCG-infected EpCAM+/ABCG2+ CSCs induces bystander apoptosis in non-infected EpCAM+/ABCG2+ CSCs. **(A)** Trypan blue assay of non-infected EpCAM+/ABCG2+ CSCs after treatment with BCG-CM or m18b-CM (CM of BCG/Mtb-m18b–infected EpCAM+/ABCG2+ CSCs grown for 12 days). **(B, C)** Cleaved caspase levels (ELISA) following 48 h of treatment with BCG-CM or BCG + Rif + CM [CM of BCG-infected EpCAM+/ABCG2+ CSCs treated with Rifampicin (Rif)]. **(D)** *In vivo* growth of tumor in a SCC-25–derived xenograftmodel of NOD/SCID mice (n = 10 in each group) treated with BCG-CM Arrow: intra-tumor treatment with 0.1 ml of sterile concentrated CM containing 0.5 mg of protein. **(E, F)** The clonogenic potential and percentage of EpCAM+/ABCG2+ CSCs in dissociated tumor cells obtained from the xenografts of 10th week after the BCG-CM treatment. Data represent means ± SEM **(B–D)**. N = 3 independent experiments **(A–C)**; N = 5 independent experiments **(E–F)**. **p < 0.01, ***p < 0.001, and ****p < 0.0001 (Student’s t-test).

Next, we evaluated the potential induction of bystander apoptosis by the CM of BCG and *Mtb-m18b–*infected EpCAM+/ABCG2+ CSCs. On day 12, the CM of BCG- or *Mtb-m18b*–infected EpCAM+/ABCG2+ CSCs were collected, filter-sterilized with 0.2-µm filter, concentrated with Centricon centrifugal filter units (EMD Millipore) to 10×, and then used to treat fresh EpCAM+/ABCG2+ CSCs. The CM-treated cells were evaluated for the *Mtb*-CFUs, cell viability, and caspase-1 and -3 protein levels. The 72-h CM-treated fresh EpCAM+/ABCG2+ CSCs showed four- to five-fold reduction in cell viability (p < 0.0001; **Figure 3A**), without any evidence of *Mtb*-CFU growth. The loss of cell viability was associated with 10- to 12-fold increase in cleaved caspase-3 level, whereas the level of cleaved caspase-1 remained unchanged (**Figure 3B**). These results suggested the induction of bystander apoptosis by the CM of BCG and *Mtb-m18b*–infected EpCAM+ABCG2+ CSCs.

As per our hypothesis, bystander apoptosis is the result of alarm signals released by the host cells, where pathogen replicates. Therefore, inhibition of pathogen replication in the host cells may significantly reduce bystander apoptosis of neighboring cells. To test this hypothesis, the BCG-infected EpCAM+/ABCG2+ CSCs were treated with rifampicin (RIF) (2 µg/ml) for 2 days that kills intracellular BCG (16, 18). On day 12, the CM (henceforth known as BCG + Rif-CM) was collected, filter-sterilized, and added to freshly grown EpCAM+/ABCG2+ CSCs. The BCG + Rif-CM–treated cells were then evaluated for cell viability and cleaved caspase-1 and -3 levels. BCG-CM–treated EpCAM+/ABCG2+ CSCs were used as positive control. We found that the BCG + Rif-CM treatment had no significant effect on cell viability and apoptosis (**Figure 3C**). These results indicate that the replication of BCG intracellular to EpCAM+/ABCG2+ CSCs is required for the CM of these cells to induce bystander apoptosis.

Next, we evaluated the *in vivo* potency of bystander apoptosis of EpCAM+/ABCG2+ CSCs in a SCC-25–derived xenograft model of NOD/SCID mice (n = 7), which we recently characterized (11). The BCG-CM, BCG + Rif-CM, or saline (1 ml/week/i.p.) were injected to SCC-25 tumor-bearing NOD/SCID mice (n = 10 in each group; initial tumor size of 0.5 mm^3^; **Figure 3D**). Tumor growth was measured weekly until the control tumor (the group with saline alone treatment) reaches the maximum size of 2cc (**Figure 3D**). At the end of the treatment, tumors were dissociated to obtain single-cell suspension; the cells were subjected to clonogenic assay, as well as immunomagnetic sorting to obtain the EpCAM+/ABCG2+ sub-population cells enriched in TSD phenotype (11) and their cleaved caspase-3 levels. Results are given in **Figures 3D–F**. We found a four-fold decrease in the tumor volume after 5 weeks of treatment in the BCG-CM versus BCG + Rif-CM–treated group (**Figures 3D, E**). Importantly, in the clonogenic assay, the EpCAM+/ABCG2+ CSC subpopulation exhibited a 15-fold reduction in the BCG-CM–treated group (**Figure 3F**). Similar findings of CM treatment that induced 10- to 15-fold reduction in EpCAM+/ABCG2+ CSC population were noted in the SK-N-BE2, RH4, H-146, and LOVO cell line–derived xenografts (data not shown). Due to the low number of EpCAM+/ABCG2+ CSCs in the CM-treated xenografts, we could not measure the caspase-3 level in these cells. Nevertheless, these results indicate the ability of the BCG-CM to target the CSC population *in vivo*.

### Intrinsic apoptotic pathway is involved in bystander apoptosis of EpCAM+/ABCG2+ CSCs

**Figure 4:**
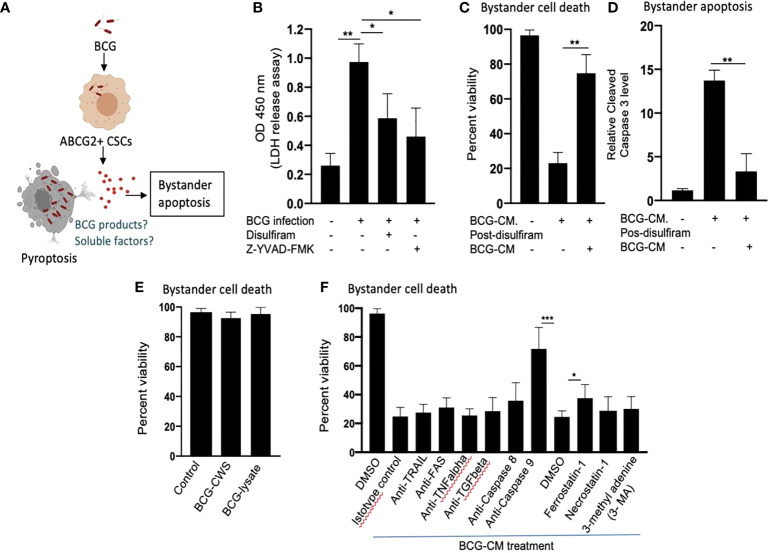
Pyroptosis-mediated secretion of soluble factors may induces bystander apoptosis. **(A)** The schematic is showing the experimental hypothesis. **(B)** The histogram is showing LDH release by BCG-infected EpCAM+/ABCG2+ CSCs on day 12. The LDH was measured after treating the BCG-infected EpCAM+/ABCG2+ CSCs with or without disulfiram and Z-YVAD-FMK from days 8 to 12. **(C, D)** Data show the uninfected EpCAM+/ABCG2+ CSC viability and bystander apoptosis following treatment of BCG-CM obtained from the infected EpCAM+/ABCG2+ CSCs with or without disulfiram treatment. **(E)** EpCAM+/ABCG2+ CSCs are not sensitive to BCG cell wall skeleton (BCG-CWS) and BCG-lysate treatment for a week. **(F)** EpCAM+/ABCG2+ CSCs were treated with various neutralizing antibodies and inhibitors during BCG-CM treatment. N = 3 independent experiments for **(B–D)**, and n = 4 independent experiment for **(E, F)**. One-Way ANOVA **(B)** and Student’s t-test **(C, D, F)**.*p < 0.05, **p < 0.01, and ***p < 0.001 (**B, F**: one-way ANOVA with Dunnet post hoc test; **C, D**: Student’s t-test).

Next, we investigated the cellular and molecular mechanisms of BCG-CM–induced bystander apoptosis including, the possibility of altruistic cell death (16, 28). We have considered that the BCG infection–induced pyroptosis may have released putative alarm signals capable of inducing bystander apoptosis in neighboring CSCs (**Figure 4A**). Indeed, BCG infection was associated with LDH release by EpCAM+/ABCG2+ CSCs (**Figure 4B**). LDH release is a marker of pyroptosis (21). Treatment of the BCG-infected cells with disulfiram (50 nM/twice daily for 4 days starting on day 8) or caspase-1 inhibitor, which are inhibitor of pyroptosis, led to a marked reduction in LDH release (**Figure 4B**). Importantly, the BCG-CM collected from the disulfiram-treated cells showed marked reduction in bystander apoptosis (**Figures 4C, D**), further suggesting that pyroptosis played a significant role in bystander apoptosis by releasing soluble factors.

We therefore considered identifying the soluble factors released by pyroptotic cells involved in bystander apoptosis. We first speculated that BCG cell wall skeleton (BCG-CWS) and/or BCG-derived DNA/RNA may be released by pyroptotic cells, thus presenting in the BCG-CM and mediating bystander apoptosis. Previously, these BCG-derived products (BCG-CWS or BCG-derived DNA/RNA) were found to induce apoptosis in bladder cancer cells by activating TLR2, TLR4, TLR7, and TLR9 and thus inducing the myeloid differentiation primary response gene 88 (MYD88) pathway of extrinsic apoptosis (26, 29). However, BCG-CWS (26) and the live-BCG lysate (1 × 10^7^ BCG in 1 ml of DMEM incubated for 24 h, and then sterile-filtered) failed to induce bystander apoptosis in EpCAM+/ABCG2+ CSCs (**Figure 4E**), suggesting that BCG-derived products were not involved in PIBA.

Next, we considered the potential release of soluble factors such as TNF-related apoptosis-inducing ligand (TRAIL), FAS ligand, TNF-alpha, and TGF-beta in the BCG-CM. These soluble factors may induce apoptosis by activating caspase-8–mediated extrinsic apoptosis (30). Previously, Kelly et al., while studying the mechanism of BCG-induced bystander apoptosis in macrophages and T cells, did not identify soluble alarm signals for bystander apoptosis (31). Similarly, we also did not find any significant role of these soluble factors in bystander apoptosis; treating the BCG-CM with neutralizing antibodies of these soluble factors did not reduce bystander cell death (**Figure 4F**). Moreover, inhibition of caspase-8 did not affect bystander apoptosis (**Figure 4F**), suggesting that extrinsic pathway was not involved in bystander apoptosis. Thus, it is unlikely that soluble factor-mediated extrinsic apoptosis was involved in BCG-CM–mediated bystander apoptosis.

In contrast, inhibition of intrinsic apoptosis, which is caused by caspase-9 (32), markedly reduced bystander apoptosis (**Figure 4F**). Thus, it appears that BCG-CM may contain soluble factors that may internalize into the target cancer cells to induce intrinsic apoptosis. We also considered the potential involvement of other mode of cell death including necroptosis, ferroptosis, and autophagy in bystander apoptosis by pretreating EpCAM+/ABCG2+ CSCs with a RIP1 kinase inhibitor (necrostatin-1), ferroptosis inhibitor (ferrostatin-1), and an autophagy inhibitor (3 MA) before treatment with BCG-CM. Relative to vehicle control (DMSO), ferrostatin-1 prevented the effect of BCG-CM–induced loss of cell viability (**Figure 4F**). Conversely, the phenotype was not reversed by necrostatin-1 or 3MA, indicating that necroptosis or autophagy does not affect the ability of BCG-CM to induce death of EpCAM+/ABCG2+ CSCs. Taken together, BCG-CM may contain soluble factors that internalize into EpCAM+/ABCG2+ CSCs to activate intrinsic apoptosis pathways.

### Bystander apoptosis of EpCAM+/ABCG2+ CSCs is associated with the release of HMGB1/p53death signal

Previously, we identified an intrinsic pathway of apoptosis mediated by p53/MDM2 oscillation (28) and HMGB1 (23) in the ASC phenotype (16, 28). HMGB1, a Damage-Associated Molecular Patterns (DAMP) associated with alarm signaling of innate defense (23, 33), is actively secreted by cancer cells during stress including hypoxia (33). Moreover, HMGB1 is secreted by BCG-infected immune cells. The extracellular HMGB1 regulates inflammation and play a pro-tumorigenic role (33). Therefore, it is unlikely that HMGB1 alone would induce bystander apoptosis of CSCs. Previously, it was found that in a colon cancer cell line, HMGB1 binding to p53, along with reactive oxygen species (ROS) production may induce apoptosis and autophagy (34). Nuclear magnetic resonance (NMR) spectroscopy and *in silico* protein structural analysis indicate that p53 can bind to HMGB1 to make stable HMGB1/p53 complex (35). Thus, it is possible that pyroptotic cells may release death signal, the HMGB1/p53 complex that mediate pathogen induced bystander apoptosis (PIBA) of neighbor CSCs in the TME.

**Figure 5:**
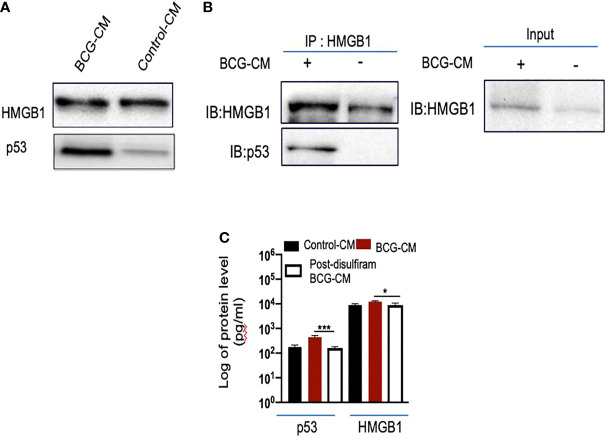
BCG-infected EpCAM+/ABCG2+ CSCs release HMGB1/p53 complex during pyroptosis. **(A)** Western blot of concentrated BCG-CM and control-CM showing the presence of HMGB1 and p53. 10 µg of protein was loaded in both infected and control group. **(B)** Immunoprecipitation experiment confirms the formation of HMGB1/p53 complex in BCG-CM versus control-CM. Immunoblotting (IB) of HMGB1 and p53 was also performed. Input is 2.5% of the total amount of immunoprecipitated. **(C)** The histogram is showing the secretion of HMGB1/p53 complex by BCG-infected EpCAM+/ABCG2+ CSCs with or without disulfiram treatment (50 nM/twice daily for 4 days). The elute of IP/HMGB1 shown in **(B)** was subjected to ELISA, and protein levels were compared with uninfected EpCAM+/ABCG2+ CSCs (p53: average, 0.13 ng/ml; HMGB1: average, 10.5 ng/ml) to obtain fold change. Data represent means ± SEM. N = 3 independent experiments **(A–C)**. *p < 0.05 and ***p < 0.001 (Student’s t-test).

To investigate the potential role of HMGB1/p53 complex in PIBA, we did a series of experiments. First, we measured HMGB1 and p53 protein levels in the BCG-CM by ELISA using iMark Microplate Absorbance Reader (Bio-Rad, Gurgaon, India) and WB assay. Next, BCG-CM was subjected to co-IP of p53 and HMGB1 to identify the putative HMGB1/p53 complex. The CM of freshly obtained uninfected EpCAM+/ABCG2+ CSCs served as a control. The results are given in **Figure 5**, showing that p53 protein could be detected in the BCG-CM only. Whereas HMGB1 could be detected in the CM of both BCG-infected and also the control group (**Figure 5A**). However, the IP-WB result demonstrates that only the BCG-CM showed the presence of a HMGB1/p53 complex, as the IP product of HMGB1 contained p53 (**Figure 5B**). Second, pretreatment with disulfiram significantly inhibited the secretion of HMGB1/p53 complex by the BCG-infected EpCAM+/ABCG2+ CSCs (**Figure 5C**). These results indicate that HMGB1/p53 complex may be secreted in BCG-CM during pyroptosis.

**Figure 6:**
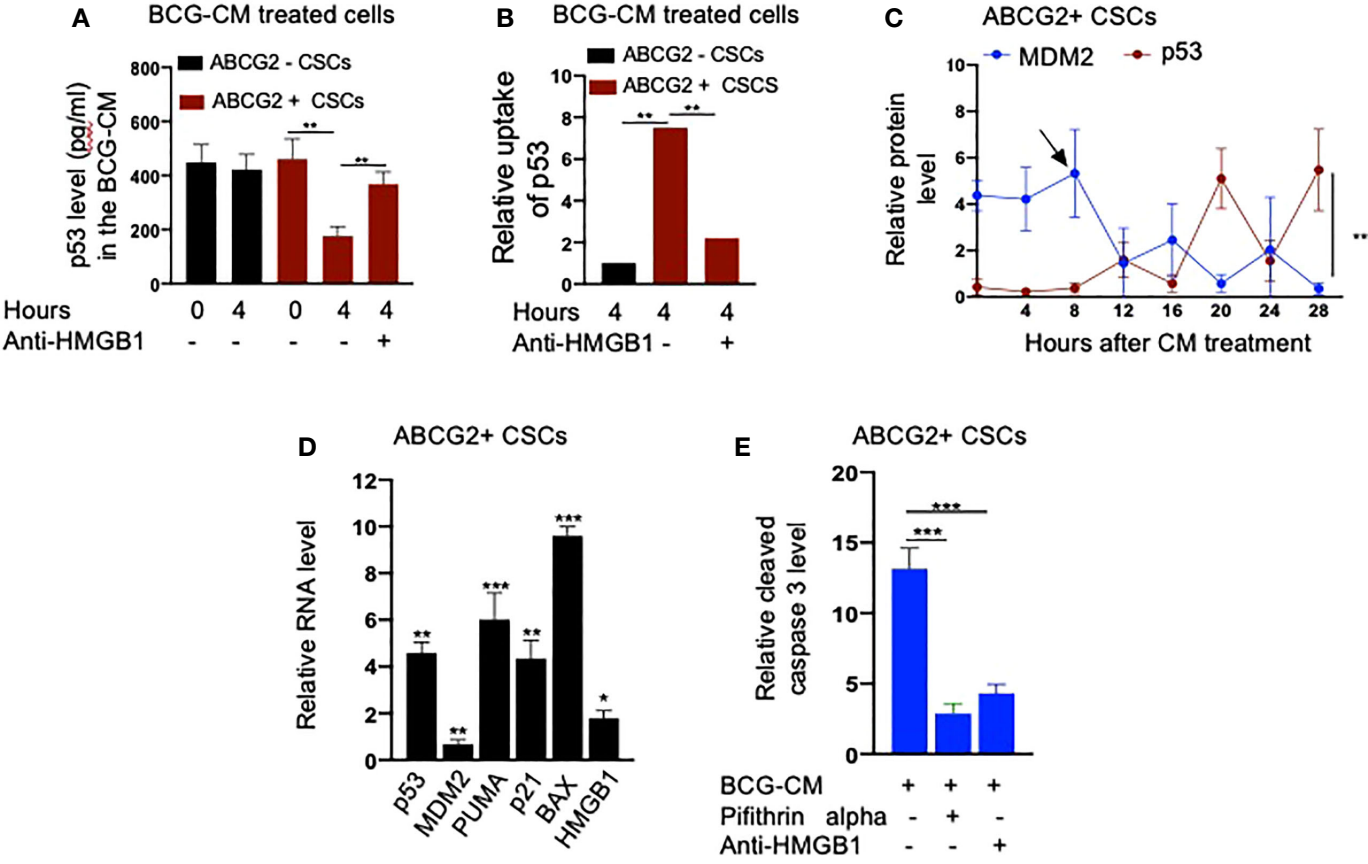
Bystander apoptosis is characterized by the HMGB1/p53 complex–mediated apoptosis. **(A)** p53 uptake by the EpCAM+/ABCG2+ versus EpCAM+/ABCG2- CSCs with or without anti-HMGB1 pre-treated BCG-CM. **(B)** Relative uptake of p53 by the EpCAM+/ABCG2+ and EpCAM+/ ABCG2- CSCs with or without neutralizing anti-HMGB1 pre-treated BCG-CM. **(C)** The induction of p53/MDM2 oscillation in EpCAM+/ABCG2+ CSCs following 12 h of BCG-CM treatment. **(D)** Significant induction of p53-related pro-apoptotic genes and HMGB1 gene in EpCAM+/ABCG2+ CSCs following 28 h of BCG-CM treatment. The real-time PCR data were compared with untreated EpCAM+/ABCG2+ CSCs to obtain fold change. **(E)** Significant increase in cleaved caspase-3 protein level in EpCAM+/ABCG2+ CSCs treated with BCG-CM with or without pifithrin alpha (2 µM in DMSO for 48 h) or anti-HMGB1 (10 µg/ml for 48 h; isotype control of same dose). Data represent means ± SEM **(A–E)**. N = 3 independent experiments **(A–E)**. *p < 0.05, **p < 0.01, and ***p < 0.001 (**A, B, E**: one-way ANOVA with Dunnet post hoc test; **C, D**: Student’s t-test).

Next, we performed a protein uptake assay to quantify the potential uptake of the HMGB1/p53 complex by the BCG-CM–treated EpCAM+/ABCG2+ CSCs. The EpCAM+/ABCG2- CSCs served as control. Briefly, p53 was measured in the CM of these cells after they were treated with BCG-CM. Reduction of the p53 concentration in the CM may indicate uptake of this protein by the cells. In this manner, we found that, within 4 h of treatment, EpCAM+/ABCG2+ CSCs took 50% of p53 from the BCG-CM (**Figure 6A**), whereas EpCAM+/ABCG2- CSCs took only 6.5%. Thus, there is a 7.5-fold increase of uptake of p53 by EpCAM+/ABCG2+ CSCs compared with EpCAM+/ABCG2- CSCs (**Figure 6B**). There was a corresponding decrease of HMGB1 concentration in the CM (11.2 ± 4.3 ng/ml to 8.4 ± 3.2 ng/ml; p = 0.043, n = 5), suggesting the uptake of HMGB1-bound p53 by the EpCAM+/ABCG2+ CSCs from the CM. Pretreatment of BCG-CM with a neutralizing antibody against HMGB1 (5 µg/ml/6 hours on ice) significantly reduced the p53 uptake by the treated cells (**Figures 6A, B**), suggesting that EpCAM+/ABCG2+ CSCs endocytose HMGB1-bound p53.

Finally, we confirmed the induction of p53/MDM2 oscillation in the BCG-CM–treated EpCAM+/ABCG2+ CSCs by performing ELISA as previously described (28). The corresponding activation of p53 downregulating genes and the increased level of cleaved caspase-3 in the BCG-CM–treated EpCAM+/ABCG2+ CSCs were also confirmed (**Figures 6C–E**). The cleaved caspase-3 level was markedly reduced following inhibition of p53 by small molecule inhibitor pifithrin alpha (**Figure 6E**) or siRNA gene silencing (16, 28), or inhibiting the HMGB1 by neutralizing antibody (**Figure 6E**). These findings suggest that the soluble factor HMGB1/p53 complex present in the BCG-CM may be associated with the bystander apoptosis in EpCAM+/ABCG2+ CSCs.

### Toll-like receptors 2 and 4 are involved in HMGB1/p53 complex–mediated bystander apoptosis

**Figure 7:**
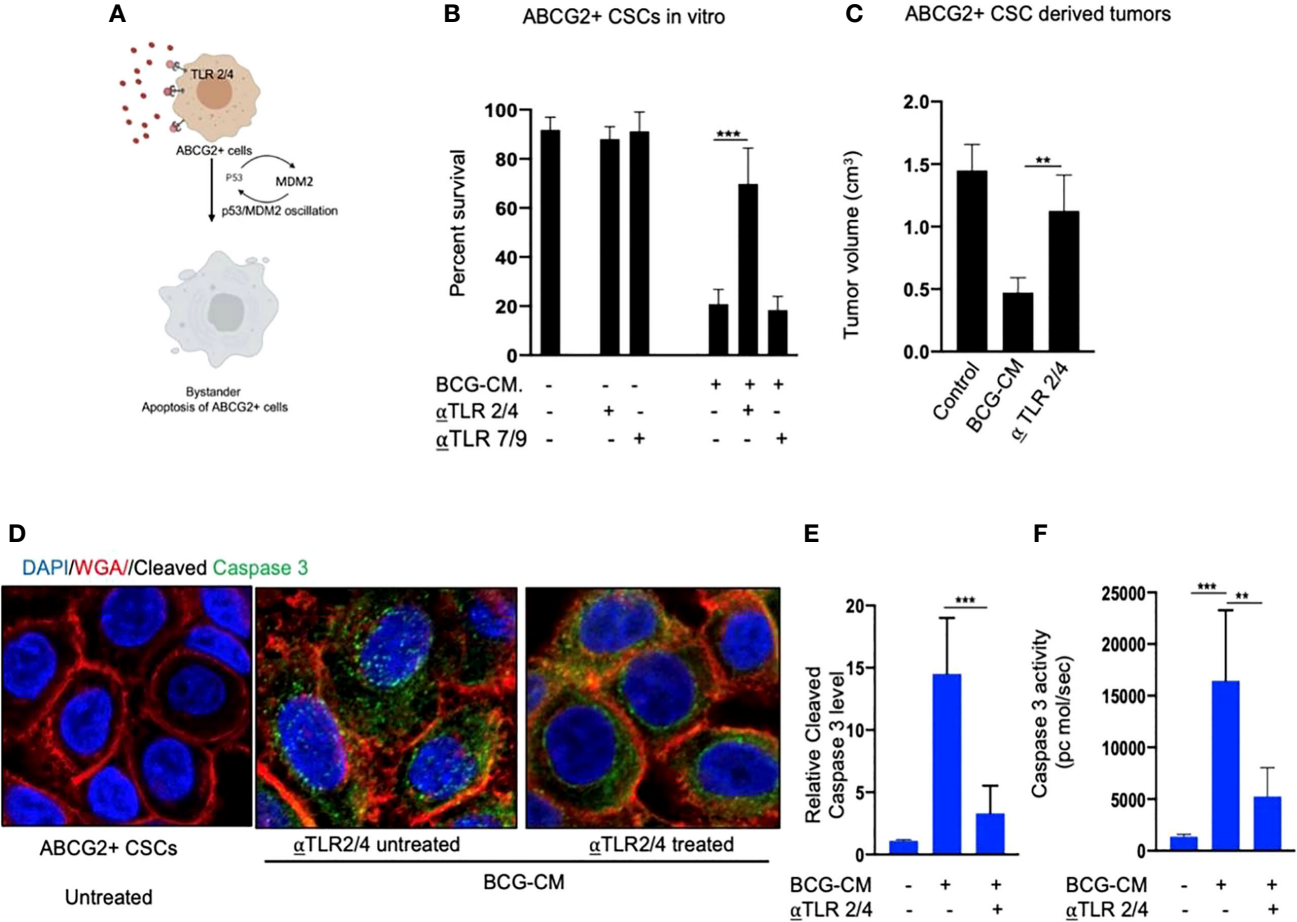
Bystander apoptosis is mediated by TLR2 and TLR4. **(A)** Hypothesis: TLR2/4 are required for the execution of HMGB1/p53 complex–mediated bystander apoptosis. **(B)** Relative cell viability of BCG-CM–treated (72 h) EpCAM+/ABCG2+ CSCs pretreated with TLR-neutralizing antibodies. **(C)** SCC-25 xenograft size in BCG-CM–treated and TLR-neutralizing Abs pretreated mice (n=5). **(D)** Immunofluorescence labeling of BCG-CM–treated (72 h) EpCAM+/ABCG2+ CSCs with cleaved caspase-3 (Dapi, nuclear stain; WGA, cell membrane stain). Magnification, 20×. **(E, F)** The corresponding cleaved caspase-3 protein level (ELISA) and enzymatic activity of caspase-3 in the EpCAM+/ABCG2+ CSCs. Data represent means ± SEM **(B, D, E)**. **p < 0.001 and ***p < 0.0001; N = 3; **(B–E)** Student’s t-test; **(F)** one-way ANOVA with Dunnet post hoc test.

Next, we examined the potential mechanism of the HMGB1/p53 molecular complex uptake into the EpCAM+/ABCG2+ CSCs for the induction of bystander apoptosis. Considering that TLR2 and TLR4 are well-known receptor for exogenous HMGB1, we reasoned that these two receptors may endocytose the HMGB1/p53 complex into the cells (**Figure 7A**) (36). Indeed, neutralizing antibodies against TLR2 and TLR4, but not TLR7 and TLR9 (5 µg/ml/4 hours; *Invivo*Gen) significantly inhibited the BCG-CM–mediated apoptosis of EpCAM+/ABCG2+ CSCs *in vitro* (**Figure 7B**) and *in vivo* (**Figure 7C**). Moreover, both confocal imaging and biochemical assay showed a marked reduction in caspase-3 expression/activity in EpCAM+/ABCG2+ CSCs treated with neutralizing antibody against TLR2/4 (**Figures 7D–F**). These data suggest that TLR2/4 may be involved in BCG-CM–mediated bystander apoptosis.

**Figure 8:**
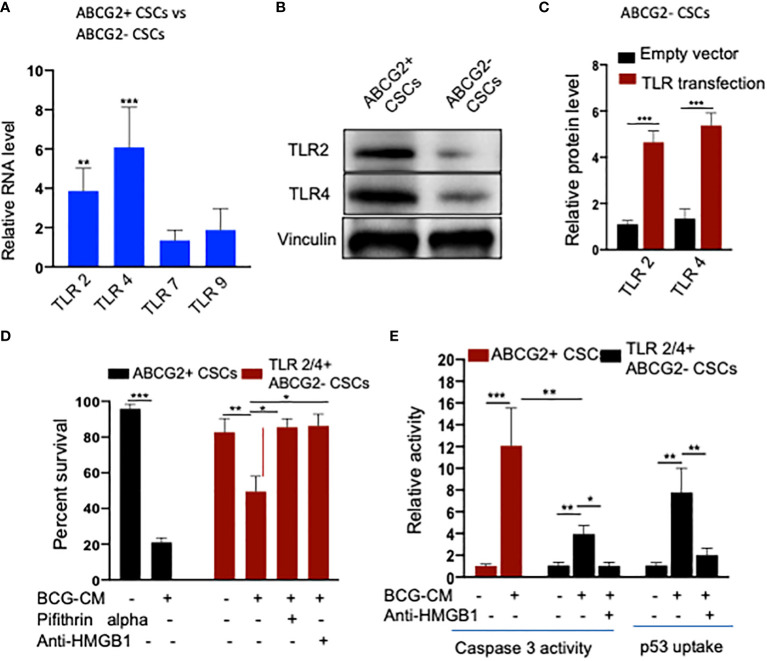
**(A)** TLR2 and TLR4 are required for the internalization of HMGB1/p53 complex into the CSCs. **(B)** Real-time PCR expression of TLR2, TLR4, TLR7, and TLR9 in EpCAM+/ABCG2+ CSCs vs. EpCAM+/ABCG2- CSCs. **(C)** Western blot shows TLR2/4 expression in EpCAM+/ABCG2+ CSCs vs. ABCG2− CSCs. **(D)** TLR2 and TLR4 plasmid transfection efficiency in EpCAM+/ABCG2- CSCs measured by ELISA. Control transfection was achieved using a pcDNA3 empty vector as described (1). **(D, E)** TLR2 and TLR4 overexpressing EpCAM+/ABCG2- showing BCG-CM–mediated bystander cell death, caspase-3 activity, and p53 uptake EpCAM+/ABCG2+ CSCs served as control for BCG-CM potency. Caspase-3 activity was measured after 48 h, whereas p53 uptake activity was measured after 4 h of BCG-CM treatment. *p < 0.05, **p < 0.001, and ***p < 0.0001, N = 3; Student’s t-test **(A, C)**, and one-way ANOVA **(D, E)**.

To further investigate the role of TLR2/4 in the uptake of HMGB1/p53 complex, we revisited the initial finding that ABCG2− CSCs are less sensitive to BCG-CM–mediated bystander apoptosis than EpCAM+/ABCG2+ CSCs (**Figures 1A**). We reasoned that ABCG2− CSCs may express low level of TLR2/4 compared with EpCAM+/ABCG2+ CSCs, leading to the less uptake of the HMGB1/p53 complex. As expected, EpCAM+/ABCG2- CSCs expressed a four- to six-fold lower level of TLR2 and TLR4 gene as well as protein expression (**Figures 8A, B**). However, there is no significant difference in expression of TLR7 and TLR9 in EpCAM+/ABCG2+ CSCs versus ABCG2− CSCs. Overexpression of TLR2/4 (**Figure 8C**) led to a two-fold increase in bystander apoptosis and an associated six-fold increase in the uptake of HMGB1/p53 complex (**Figures 8D, E**). In addition, this TLR2/4-dependent increase in bystander apoptosis is markedly reduced following inhibition of p53 or neutralizing of HMGB1 in the BCG-CM (**Figures 8D, E**). These results indicate that TLR2 and TLR4 are involved in the HMGB1/p53 complex–mediated bystander apoptosis.

### TS phenotype amplifies the BCG-CM–mediated bystander apoptosis

**Figure 9:**
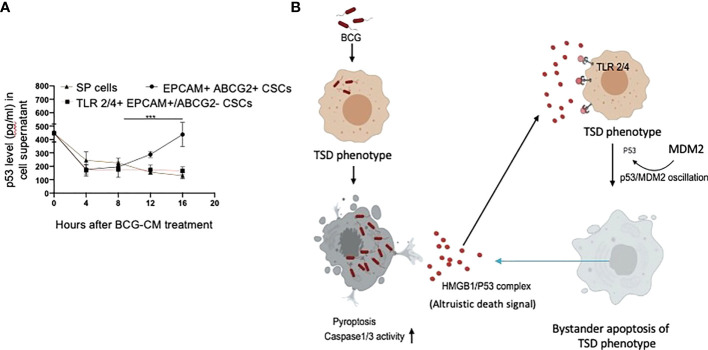
Tumor stemness defense (TSD) phenotype can amplify the pathogen-induced bystander apoptosis (PIBA). **(A)** The p53 uptake assay in the culture supernatant was measured from 0 to 16 h of BCG-CM treatment in the cells. The SCC-25 SP cells were obtained as described in **Figure 1** Data represent mean +/- SEM, n= 3 independent experiments. ***p < 0.0001, student t test. **(B)** Potential mechanism of TSD phenotype–mediated niche defense of CSCs against BCG infection. In the infected CSCs, as part of the Altruistic stemness–based (37) niche defense mechanism (6, 16, 17) HMGB1 form a complex with cytoplasmic p53 to make an, “altruistic death signal”. TLR4 internalizes the altruistic death signal, leading to induction of p53/MDM2 oscillation and activation of p53-induced pro-apoptotic genes. The EpCAM+/ABCG2+ CSCs undergoing bystander apoptosis further release the HMGB1/p53 death complex into the TME, amplifying PIBA.

We noted that bystander apoptosis was significantly less in TLR2/4-overexpressing EpCAM+/ABCG2-CSCs than the EpCAM+/ABCG2+ CSCs (**Figure 8E**), although the HMGB1/p53 (present in the BCG-CM) uptake was similar (**Figures 6B**, **8E**). We hypothesize that, in the EpCAM+/ABCG2+ CSCs that are of TS phenotype, the HMGB1/p53 apoptotic signal may be amplified as a part of the CSC niche defense mechanism. Thus, we expect that the p53 concentration in the CM of EpCAM+/ABCG2+ CSCs will increase after initial decline due to the fresh release of HMGB1/p53 complex from the cells undergoing bystander apoptosis. Whereas in the SP cells or and TLR2/4 overexpressing EpCAM+/ABCG2- CSCs that do not exhibit TS phenotype, the death signal would not be amplified. Indeed, we found that the p53 concentration in the EpCAM+/ABCG2+ CSCs exhibited a sharp increase by 2.5-fold between 8 and 16 h of BCG-CM treatment after initial decline in 4 h, suggesting the release of fresh HMGB1/p53 complex by the apoptotic cells. Whereas the p53 concentration in the culture supernatant of SP cells and TLR2/4 overexpressing EpCAM+/ABCG2- CSCs did not increase between 8 and 16 h of BCG-CM treatment (**Figure 9A**). Moreover, we did the co-IP assay of EpCAM+/ABCG2+ CSCs that showed increase in HMGB1-bound p53 by three-fold (160 ± 22 ng/ml versus 485 ± 32 ng/ml; n = 4; p = 0.02) between 8 and 16 h of BCG-CM treatment (data not shown). These data further confirm the release of fresh HMGB1/p53 complex by the BCG-CM–treated EpCAM+/ABCG2+ CSCs. Importantly, the culture supernatant containing this high level of HMGB1/p53 complex further induced bystander apoptosis to another untreated population of EpCAM+/ABCG2+ CSCs (data not shown), suggesting amplification of the original alarm/death signal **Figure 9B**.

## Discussion

CSCs promote invasion, metastasis, and drug resistance (36, 38–44). CSCs may reside in the hypoxic niche (5, 45), and reprogram to a highly aggressive phenotype (46–52) TS switch (TSS) phenotype in the TME of oxidative stress (5–7, 28, 53–55). Targeting CSCs in the hypoxic niche with the existing therapeutic strategies such as targeting CSC surface markers (56), signaling cascades like Notch, Hedgehog, Wnt, and nuclear factor kappa B (NF-κB) (57) as well as multikinase inhibitors (58–60) are not successful (61, 62). Here, we demonstrate that post-hypoxia SPm (hox)+/ABCG2+ CSCs of seven cancer cell lines including SCC-25 exhibit a TSD phenotype against BCG and Mtb-m18b that involve bystander apoptosiss and therefore could be of potential use to eliminate CSCs. Mechanistic study done in the post-hypoxia/reoxygenation EpCAM+/ABCG2+ CSCs of SCC-25 infected with BCG revealed that these cells undergoes pyroptosis and release the HMGB1/p53 complex. This complex serves as a death signal and induces p53-mediated apoptosis of bystander EPCAM+ ABCG2+ and ABCG2− CSCs in a TLR2- and TLR4-dependent manner. Importantly, the death signal–rich CM exert significant toxicity to EPCAM+ABCG2+ CSCs residing in the hypoxic niche of tumor xenografts. Thus, our work indicates that pathogen-infected CSC of TS phenotype releases a death signal that eliminates the nearby CSCs. This putative CSC niche defense may have potential significance as a novel strategy to target CSCs in their hypoxic niche.

Our work has unraveled a unique functional role of the hypoxia/reoxygenation enhanced TS phenotype of CSCs, the defense against intracellular pathogen such as BCG or Mtb-m18b. Thus, it appears that CSCs reprogram to this stemness state to defend their TME niche not only against oxidative stress or chemotherapy-induced stress (5–8) but also against pathogen invasion. Therefore, this CSC phenotype, which we previously termed as the TSS phenotype (5–8), can be better way termed as the TSD phenotype. We suggest the bystander apoptosis mediated by BCG-infected TSD phenotype is a form of putative “CSC niche defense” mechanism similar to Altruistic Stemness (37) Defense (ASD) phenotype–mediated “stem cell niche defense” mechanism (7, 16), (**Figure 9B**) that we recently described in virus-infected MSCs (16) and hypoxia/oxidative stress–exposed human ES cells (28).

One shared characteristic of TSD and ASD phenotype–mediated defense is PIBA, which is a part of the innate immune defense mechanism that protects host cells from invading pathogens (63). Kelly *et al.* reported *Mycobacteria-*mediated PIBA in macrophages, where *Mtb*-infected macrophages exert PIBA by direct cell to cell contact (31). The HIV-infected CD4+ T cells exert PIBA, which is mediated by the viral envelope protein (64). We speculated that PIBA may be involved in stem cell niche defense mechanism, which we recently reported in virus and *Mtb-m18b*–infected lung alveolar MSCs derived ASCs (16). Thus, bystander apoptosis may represent a stem cell niche defense mechanism against pathogen invasion in the niche. CSCs may hijack this defense mechanism to protect their TME niches from invading pathogens. Considering the growing evidence of tumor-associated microbes and their role in tumor growth/metastasis, the idea and evidence of TSD phenotype–mediated defense presented here may have broader implications in the growing field of tumor microbiome. Further studies of the potential role of TSD phenotype in defense against immunotherapy, radiation therapy, and chemotherapy may provide better insight on the mechanism that underlies inherent competence of CSCs to persist despite therapies.

The TSD-mediated defense may be a two-edged sword: Although the defense may protect CSCs from oxidative stress or chemotherapy as reported earlier (5–7), the defense may induce bystander apoptosis to protect the TME from invading pathogens.

In this context, BCG being FDA-approved immunotherapy in invasive bladder cancer, we wanted to explore a putative BCG-induced bystander apoptosis of TSD phenotype. The *Mtb-m18b* strain that showed activation of stem cell niche defense by MSCs derived ASCs (16) served as a positive control. Thus, the post-hypoxia/oxidative stress [SPm (hox)+/ABCG2+ CSCs of several cell lines that we previously characterized for the TS phenotype SPm (hox) enriched in EpCAM+/ABCG2+ CSCs], including oral cancer, breast cancer, and lung cancer, were infected with *Mtb-m18b* and BCG. We found that the pathogen replicated intracellular to EpCAM+/ABCG2+ CSCs. The CM of these cells showed anti-bacterial activity against Mtb-18b and bystander cell death of freshly obtained non-infected EpCAM+/ABCG2+ CSCs. Whereas the pathogens did not infect and replicate intracellular to EpCAM+/ABCG2- CSCs (**Figure 2**); CM of these cells did not induce bystander apoptosis (**Figure 1**), suggesting that PIBA is limited to TS phenotype only. We then studied the molecular mechanism of PIBA of post-hypoxia/oxidative stress EpCAM+/ABCG2+ CSCs of SCC-25 cell line. We found that the BCG-treated EpCAM+/ABCG2+ versus ABCG2-CSCs caspase-1 and gasdermin D on day 12 (**Figure 2**) underwent pyroptosis; the CM of these cells contained HMGB1/p53 death signal that induced p53-mediated apoptosis of the uninfected CSCs in a TLR2/4-dependent manner. The p53 activation was preceded by the induction of p53/MDM2 oscillation, a mode of cell death observed in ASCs (16, 28). Notably, the treatment of infected EpCAM+/ABCG2+ CSCs with disulfiram (an inhibitor of pyroptosis) failed to induce bystander apoptosis. These results suggested that during pathogen-induced pyroptosis, the dying cells release HMGB1/p53 death signal complex that induces bystander apoptosis in neighboring EpCAM+/ABCG2+ and ABCG2− CSCs. Subsequent findings suggest that only the EpCAM+/ABCG2+ CSCs can amplify the HMGB1/p53 death signal, i.e., cells undergoing bystander apoptosis can release HMGB1/p53death signal, whereas EpCAM+/ABCG2- CSCs undergoing bystander apoptosis failed to releaseHMGB1/p53 complex into the culture supernatant. This may explain the reason of low level of bystander apoptosis in this cell phenotype (**Figure 1**).

HMGB1 has a potent immuno-suppressive (65) and pro-survival properties as it activates NF-κB *via* TLR signaling pathways. However, HMGB1 may also induce apoptosis or autophagy by forming a complex with p53, as reported in a human colon cancer cell line HCT116 (34). The nature of the HMGB1 and p53 binding is not yet clear but may form a stable complex, as previously shown (35). Whereas the HMGB1/p53 complex may mediate altruistic cell death in the human embryonic stem cells (hESCs) and MSC-derived ASC phenotype by the activation of the p53/MDM2 oscillation (16, 23, 28). We consider this altruistic cell death as an important component of the putative ASC-based stem cell niche defense mechanism (16, 28). In this context, our findings of BCG-CM–mediated release of HMGB1/p53and the subsequent activation of the p53/MDM2 oscillation in the ABCG2+ CSCs may be viewed as a part of the ASC-based stem cell niche defense mechanism being activated by the CSCs. Whereas EpCAM+/ABCG2- CSCs undergoing bystander apoptosis failed to release HMGB1/p53 complex into the culture supernatant and therefore failed to activate the ASC-based stem cell niche defense mechanism.

TLR2 and TLR4 may be involved in this putative niche defense mechanism as the inhibition of these two receptors significantly reduced bystander apoptosis of EpCAM+/ABCG2+ CSCs. The TLRs are an integral part of innate immune defense mechanism. However, in cancer, TLRs act as a double edged sword in either favoring stemness (66, 67), or apoptosis (68). BCG activates TLR2/4 in foam macrophages and T cells to induce metabolic reprogramming (69), and T-cell response (70). In the bladder cancer cells, BCG was shown to induce apoptosis *via* TLR7 (71). However, in our study, we found the involvement of TLR2/4, but not TLR7 in the PIBA of EpCAM+/ABCG2+ CSCs. TLR2 and TLR4 are surface receptors, and their cellular signaling mechanisms are mediated primarily by MYD88 adaptor protein (66, 67). TLR4, which is known to participate in receptor-mediated endocytosis (72) and possibly TLR2, may be capable of internalizing the HMGB1/p53 complex into the cytoplasm of CSCs (**Figure 8E**). The complex then activates a putative altruistic death signaling characterized by the induction of p53/MDM2 oscillation. The altruistic death signal is amplified by TSD phenotype undergoing bystander apoptosis (**Figure 9B**). Future studies are required to unravel the role of TLR2 and TLR4 in the internalization of HMGB1/p53 complex into the EpCAM+/ABCG2+ CSCs and in the subsequent induction of p53/MDM2 oscillations.

Taken together, our mechanistic study of BCG-induced bystander apoptosis of TSD phenotype indicates a mode of cell death similar to the cell death of ASD phenotype of embryonic stem cells (28) and MSCs (16). Further investigation is required to determine whether the activation of ASC-based stem cell niche defense mechanism underlies marked anti-tumor activity observed in the *in vivo* xenograft assay (**Figure 3D**). One promising therapeutic aspect of the ASC-based niche defense mechanism is the altruistic death that involves the activation of p53/MDM2 oscillation in CSCs. During the last two decades, various strategies have been explored to activate p53 in tumor cells, including small molecules that can disrupt MDM2, inhibit nuclear translocation, and/or reverse mutant to wild-type conformation (73). However, none of these mechanisms involve the induction of p53/MDM2 oscillation. In this context, further investigation of the downstream of TLR2/4 mediated activation of p53/MDM2 oscillation in EpCAM+/ABCG2+ CSCs may provide new ways to activate p53 in tumor cells. We speculate that the suppression of p53 inhibitory stemness pathways such as MYC–hypoxia-inducible factor 2α (HIF-2α) stemness pathway may be involved in the activation of p53/MDM2 oscillation. Using EU-MYC model of T-cell acute lymphoblastic leukemia, we reported that SCA1+ CSCs use MYC–HIF-2α stemness pathway to suppress p53 and decrease ROS production (1). The MYC–HIF-2α stemness pathway is also active in the EpCAM+/ABCG2+ CSCs of SCC-25 cell line (11, 13, 23). In hESCs derived ASCs, the HIF-2αstemness pathway was activated; inhibition of the pathway led to the induction of p53/MDM2 oscillation (28). Whether the BCG-CM–mediated p53/MDM2 oscillation of EpCAM+/ABCG2+ CSCs is the result of inhibiting the MYC–HIF-2α stemness pathway is now under active investigation.

The prospective contribution of this work in the field of targeting CSCs needs further investigations. Although our study demonstrated the potential of ASC-based defense as a novel therapeutic strategy against CSCs, several limitations should be considered. First, the efficacy of this therapeutic strategy in an *in vivo* immunocompetent tumor model is not known. Furthermore, most of our work has been performed *in vitro*, using hypoxia/oxidative stress–enhanced side-population cells or EPCAM+/ABCG2+ cells of established tumor cell lines. The SP cells are heterogeneous and contain both rapidly cycling EpCAM+/ABCG2+ and quiescent or primitive ABCG2− cells (25, 74). SP fraction is now generally applied to indicate stemness (75, 76, 79), and TME stress mediated drug resistance resistance (77–81). In this context, the selective targeting of CSCs exhibiting only the TSD phenotype may limit the anti-tumor efficacy in the immunocompetent tumor models. Another limitation of the study is the lack of data from primary human tumor–derived CSCs. We obtained mechanistic data from EpCAM+/ABCG2+ CSCs of SCC-25 tongue cancer cell line but did not include equivalent primary CSCs in the study. The SCC-25 obtained EpCAM+/ABCG2+ CSCs were isolated from a post-hypoxia/reoxygenation population of EPCAM+ cells expressing high levels of ALDH1, CD133, and CD44v3, markers of CSCs (11, 12) whereas the EpCAM+/ABCG2- CSC counterpart also equally expressed these cell surface markers. Thus, both EpCAM+/ABCG2+ and ABCG2− cells belongs to the heterogeneous EPCAM+/ALDH+/CD44+/CD133+ CSC population. However, only the EpCAM+/ABCG2+ but not ABCG2− CSCs showed amplification of PIBA (**Figure 9A**). Although the reason could be the TLR expression, overexpression of the TLR in the non-TSD phenotype did not lead to amplification of the death signaling (**Figure 9A**). Whether the primary tumor–derived EpCAM+/ABCG2- CSCs exhibit BCG-induced PIBA requires further investigation.

In summary, here, we provide experimental evidence that the TSD phenotype of SCC-25–derived CSCs exhibits niche defense against BCG infection. We found that the CM of BCG-infected CSCs release HMGB1/p53 complex, which then induces p53/MDM2 oscillation in uninfected CSCs of TSD phenotype (**Figure 9B**). This bystander apoptosis can be inhibited by neutralizing antibodies against HMGB1, TLR2, and TLR4, or small-molecule inhibitor of p53. We speculate that the BCG-induced bystander apoptosis is a part of the recently identified ASC-based stem cell niche defense mechanism against pathogen invasion. Understanding this TSD phenotype–mediated niche defense may help to gain insight into hypoxia and oxidative stress mediated tumor progression (6, 7, 54) tumor progression and to develop innovative strategies to target CSCs in their niches.

## Data availability statement

The raw data supporting the conclusions of this article will be made available by the authors upon reasonable request.

## Ethics statement

This study was reviewed and approved by Institutional Animal Ethics Committee of KaviKrishna Laboratory, and Gauhati University.

## Author contributions

BD initiated and designed the study. BD, SB, BP, LP, PS, SM, SG, RM and HL performed the *in vitro* and *in vivo* experiments. BD, SB, BP, and LP analyzed data. BD, SB, LP, BP and SG wrote the article. BD, LP, PS, SM, BP, CR, and DB edited the article. All authors contributed to the article and approved the submitted version.

## Funding

Funding was obtained from Laurel Foundation, USA, at Forsyth Institute, Cambridge, MA, USA (BD); the KaviKrishna Foundation (Assam, India) grants KKL/2014-1_CSC (SB, LP, PS, SM, and SG); Department of Biotechnology India grant BT/PR22952/NER/95/572/2017 (BD); and the KaviKrishna USA Foundation grants KKL/2018-2_CSC (BD, BP, and RM).

## Acknowledgments

We thank Professor Emeritus Herman Yeger of University of Toronto, Dr. Antonio Campos Neto and Dr. Philip Stashenko of (Forsyth Institute, Cambridge, MA, USA) for their valuable suggestions in this research work. We also thank Dr. Jyotirmoi Phukan (Gauhati Medical College and Hospital) and Dr. Anupam Sarma (B. Borooah Cancer Institute) for their valuable suggestions. We thank Mr. Biswajit Das and Mallika Maral of KaviKrishna Laboratory for taking care of the animal facility. We thank the members of KaviKrishna Laboratory, Indian Institute of Technology Guwahati Research Park, Guwahati, Assam, India; Thoreau Laboratory for Global Health, M2D2, University of Massachusetts, Lowell, Massachusetts; and Department of Bioengineering and Technology, Gauhati University, Guwahati, Assam, India.

## Conflict of interest

The authors declare that the research was conducted in the absence of any commercial or financial relationships that could be construed as a potential conflict of interest.

## Publisher’s note

All claims expressed in this article are solely those of the authors and do not necessarily represent those of their affiliated organizations, or those of the publisher, the editors and the reviewers. Any product that may be evaluated in this article, or claim that may be made by its manufacturer, is not guaranteed or endorsed by the publisher.
