# Supplementary material for: Targeting hypoxia-induced tumor stemness by activating pathogen-induced stem cell niche defense
Source: Front Immunol. 2022 Sep 29;13:933329. doi: 10.3389/fimmu.2022.933329 (PMC9559576; doi:10.3389/fimmu.2022.933329)
Supplement: Supplementary file 1 [file Table_1.pdf]

Bhuyan S and Pal B et al. Supplementary Table 1

Protein name	Antibody	ELISA kit
p53	2527 (Cell Signaling Technology)	DYC1043-2 R&D
MDM2		DYC1244-2, R&D
Beta-Actin	#3700 (Cell Signaling Technology)	
Vinculin	#4650 (Cell Signaling Technology)	
TLR 4	NB100-56566 (Novus Biologicals) for ELISA/WB.<br>Mabg-htlr4 (InvivoGen, San Diego, CA) for neutralizing.	
Cleaved Gasdermin D (GSDMD)	#36425 rabbit polyclonal (Cell Signaling Technology) and # H00079792-M01 (Abnova).	
TLR2	NB100-56722 (Novus Biologicals) for ELISA/WB.<br>Maba2-htlr2 (InvivoGen, San Diego, CA) for neutralizing.	
HMGB1	H00003146-M08 (Novus Biologicals, Littleton, CO) Western blot, ELISA and neutralization.<br>#ab228624 A(Abcam) for IP.	#NBP2-62766, Novus Biologicals
Cleaved caspase 1	#A1004 rabbit polyclonal (Biovision, Milpitas, CA). #sc-56036 mouse monoclonal (Santacruz)	
ABCG2	AB3380 (Abcam)	MBS703358 Mybiosource, CA
Cleaved caspase 3	PAS-114687 (Thermofisher Scientific)	DYC835-2
